# PEPTiGEN: a tool for mining antimicrobial resistance PEPTides using GENe data of public available repositories

**DOI:** 10.1093/bioinformatics/btag604

**Published:** 2026-08-18

**Authors:** Lisa M Meekes, Francesco Tabaro, Michiel L Bexkens, Dimard E Foudraine, Lennard J M Dekker, Theo M Luider, Nikolaos Strepis, Corné H W Klaassen, Wil H F Goessens

**Affiliations:** Department of Medical Microbiology and Infectious Diseases, Erasmus University Medical Center Rotterdam, Rotterdam, 3015 GD, Netherlands; Genevia Technologies Oy, Tampere, 33100, Finland; European Molecular Biology Laboratory, Epigenetics and Neurobiology Unit, Monterotondo, RM 00015, Italy; Department of Medical Microbiology and Infectious Diseases, Erasmus University Medical Center Rotterdam, Rotterdam, 3015 GD, Netherlands; Department of Medical Microbiology and Infectious Diseases, Erasmus University Medical Center Rotterdam, Rotterdam, 3015 GD, Netherlands; Department of Neurology, Clinical and Cancer Proteomics, Erasmus University Medical Center Rotterdam, Rotterdam, 3015 GD, Netherlands; Department of Neurology, Clinical and Cancer Proteomics, Erasmus University Medical Center Rotterdam, Rotterdam, 3015 GD, Netherlands; Department of Medical Microbiology and Infectious Diseases, Erasmus University Medical Center Rotterdam, Rotterdam, 3015 GD, Netherlands; Department of Medical Microbiology and Infectious Diseases, Erasmus University Medical Center Rotterdam, Rotterdam, 3015 GD, Netherlands; Department of Medical Microbiology and Infectious Diseases, Erasmus University Medical Center Rotterdam, Rotterdam, 3015 GD, Netherlands

## Abstract

**Motivation:**

Detecting antimicrobial resistance (AMR) remains challenging due to the complexity and evolution of resistance mechanisms. Liquid chromatography online coupled to tandem mass spectrometry (LC-MS/MS) offers a promising diagnostic tool. Its success, however, depends on an up-to-date database which can be used to target AMR specific peptides.

**Results:**

We present PEPTiGEN, a computational tool that automatically generates tryptic peptides for any prokaryotic gene and its variants. PEPTiGEN was validated both *in silico* and *in vitro*, showing 99% accuracy compared to manually generated tryptic peptides and 98% compared to experimental mass spectrometry data. To demonstrate its potential, we used PEPTiGEN to generate the first AMR peptide database by screening publicly available nucleotide AMR sequences using the Comprehensive Antibiotic Resistance Database (CARD). Together, PEPTiGEN and the AMR peptide database are cornerstones for advancing LC-MS/MS applications in AMR detection and clinical diagnostics.

**Availability:**

The PEPTiGEN code and AMR peptide database are publicly available at github (https://github.com/ftabaro/inspection) and Zenodo (https://doi.org/10.5281/zenodo.21196702).

## 1 Introduction

Antimicrobial resistance (AMR) is one of the top global public health and developmental threats listed by the World Health Organization (WHO) (World Health Organization 2023a). With an estimated 4.71 million global deaths associated with AMR in 2021 (GBD Antimicrobial Resistance Collaborators 2024), the WHO set research on diagnostics and treatment for AMR as a priority in 2023 (World Health Organization 2023a, 2023b). Diagnosing AMR is challenging due to the involvement of not only diverse bacterial species and sample types but also the existence of a wide array of resistance mechanisms (Darby *et al.* 2023). Most currently implemented diagnostics for AMR rely on culture-based phenotypic tests, which are inherently time-consuming (Gajic *et al.* 2022). Molecular approaches, such as PCR, are often used to complement these methods by targeting known resistance mechanisms. However, direct detection of AMR determinants without prior culture remains challenging (Gajic *et al.* 2022).

Recently, several diagnostic assays using liquid chromatography online coupled to tandem mass spectrometry (LC-MS/MS) have been validated for detecting AMR mechanisms, using both targeted and untargeted methods (Charretier *et al.* 2015a, 2015b, Alves *et al.* 2022, Foudraine *et al.* 2022, Deforet *et al.* 2024, Blumenscheit *et al.* 2026). This novel approach directly identifies proteins conferring antibiotic resistance by rapid and accurate detection of enzymatic cleaved peptides. The use of LC-MS/MS has multiple advantages over the established diagnostics for antimicrobial susceptibility testing. First, LC-MS/MS assays can give results in 1–3 h (Charretier *et al.* 2015b, Wang *et al.* 2019a, Foudraine *et al.* 2022, Deforet *et al.* 2024, Blumenscheit *et al.* 2026) in contrast to 18–48 h for conventional culture-based diagnostics (Gajic *et al.* 2022). Second, LC-MS/MS assays enable quantification of AMR-proteins in contrast to genomic-based diagnostics that just identify the presence or absence of AMR genes (Charretier *et al.* 2015b, Deforet *et al.* 2024). This allows detection of upregulation and downregulation of chromosomal resistance mechanisms which play a key role for different pathogenic bacteria, such as *Pseudomonas aeruginosa* (Charretier *et al.* 2015b, López-Causapé *et al.* 2018) and other ESKAPE pathogens (Gauba and Rahman 2023). Finally, by detecting the protein itself, LC-MS/MS provides a direct signature of an active AMR mechanism, rather than inferring activity from gene presence. As horizontally acquired resistance genes drive most prevalent mechanisms of resistance, LC-MS/MS is particularly effective for direct detection of these mechanisms.

Successful implementation of high-resolution mass-spectrometry requires an up-to-date peptide database for AMR. While genomic databases are constantly updated, no publicly available database currently catalogues AMR tryptic peptides. Despite the availability of different tools and workflows such as sequence alignment software and sequence translation tools, generating peptide information for an AMR gene remains a complex and laborious task due to the number of publicly available AMR genes. Recent publications have applied large-scale bioinformatic analyses and machine learning to publicly available genomic data to identify antimicrobial peptides as potential therapeutics (Santos-Júnior *et al.* 2024, Torres *et al.* 2024), but these approaches have not systematically targeted AMR-associated peptides. Workflows such as Bacterial Peptide Sequence Selection (BPSS) (Bastien *et al.* 2026) have demonstrated the feasibility of generating non-redundant peptide sets from large genomic datasets. Building on these approaches, we developed PEPTiGEN, a computational tool that automatically generates tryptic peptide information for any given prokaryotic gene and its variants. We validated the tool *in silico* and *in vitro* using published MS data from both our own and other research groups. To promote the adoption of LC-MS/MS in research and clinical diagnostics, we compiled all known AMR-genes from publicly available sources and used PEPTiGEN to populate a database of predicted enzymatically derived peptides. Unlike full-length protein FASTA databases, the AMR peptide database comprises tryptic peptides of MS-compatible length. This peptide-centric design facilitates efficient identification of both conserved and gene-specific peptides for targeted proteomics workflows.

## 2 Methods

### 2.1 Pipeline overview

**Figure 1:**
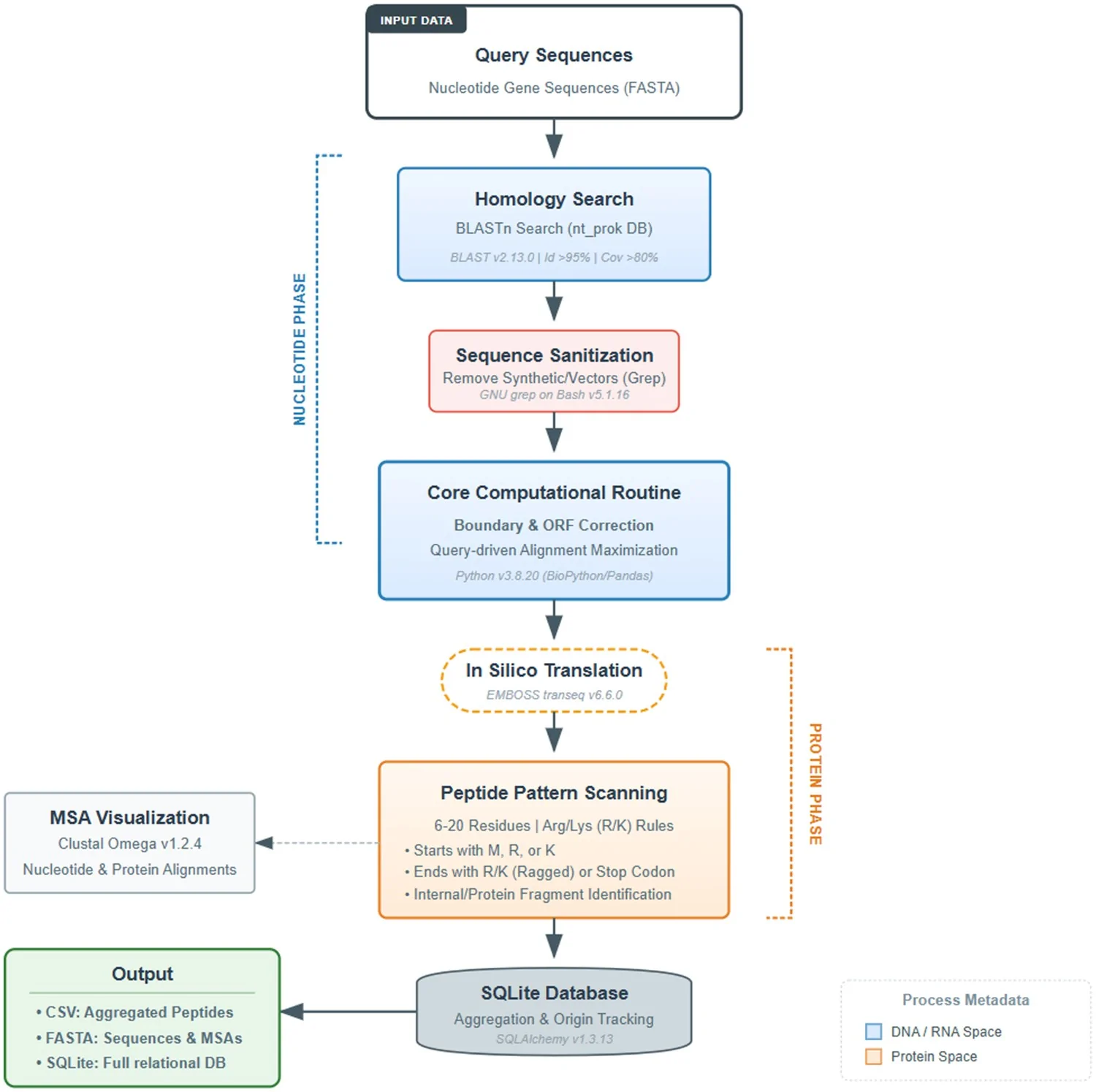
PEPTiGEN workflow overview. PEPTiGEN retrieves and processes gene variants from a user-provided nucleotide sequence by performing BLAST searches against public or local databases. Retrieved sequences are filtered, aligned, and translated *in silico* into proteins. Tryptic peptides are then generated, annotated, and stored in an SQLite database. Depending on the tool version, PEPTiGEN also aligns both nucleotide and predicted protein sequences [multiple sequence alignments (MSA)]. The workflow generates multiple outputs, including nucleotide and protein FASTA files, and a peptide table for downstream analysis.

The PEPTiGEN tool aims at predicting peptide sequences from nucleotide sequences. For each given nucleotide sequence, it performs a query against a nucleotide database looking for homologous sequences. Retrieved homologous sequences are translated *in silico* to protein sequences. Resulting protein sequences are processed to peptides by scanning for a given sequence pattern. For each predicted peptide, the tool reports GenBank entries displaying instances of that peptide sequence and two files reporting multiple sequence alignments of both nucleotide and predicted protein sequences (Fig. 1).

### 2.2 PEPTiGEN details

The tool accepts one or more gene sequences in FASTA format as input. The first step is a BLAST search (BLAST v2.13.0). End users can tune BLAST parameters before running the tool, but default values are preset and distributed with the software—80% for coverage and 95% for identity. The identity threshold is defined at the nucleotide level and, therefore, does not correspond directly to amino acid substitutions. By default, PEPTiGEN searches the nt_prok database, which contains GenBank and RefSeq nucleotide sequences stripped of eukaryotic genome assemblies. PEPTiGEN implements an automatic method to download the BLAST database. Users have the option to customize and choose the version of the target BLAST database.

BLAST results are processed to remove synthetic and artificial vector sequences (GNU grep on Bash v5.1.16). Remaining identifiers are used to query the BLAST database to retrieve their nucleotide sequences. These identifiers serve as the entry point for the core routine of the pipeline, which aims to identify peptide sequences (implemented as a Python v3.8.20 script using BioPython v1.76 and Pandas v1.5.3). This routine discards nucleotide sequences with query coverage and/or sequence identity values below user-defined thresholds. For the identifiers that meet the BLAST criteria, alignment boundaries are automatically corrected to maximize query coverage. If a partial alignment is detected, the tool corrects alignment boundary positions by extending them in the correct direction, depending on the side of the mismatch region (3′, 5′, or both), the alignment direction (forward or reverse), and the open reading frame (ORF). Finally, it extracts the corresponding sequence maintaining the original ORF.

Subsequently, the resulting nucleotide sequences are translated into amino acid sequences using transeq (EMBOSS v6.6.0) and scanned for instances of a peptide pattern. A peptide pattern was defined as an amino acid sequence with a range of 6–20 residues. The peptide can start with Methionine (first amino acid of a possible protein sequence) or one or more Arginine or Lysine residues to provide information about the variable location within the protein sequence (start of the sequence, fully contained or ragged beginning). The peptide was set to end with one or more Arginine or Lysine residues (ragged ends) or a stop codon. Any residue is allowed to compose the middle of the peptide, except for Arginine or Lysine; to omit missed cleavages. Predicted peptides are stored in a table in the internal SQLite database while keeping track of their gene of origin.

Both selected nucleotide and predicted protein sequences are aligned using Clustal Omega v1.2.4 to produce multiple sequence alignments suited for downstream analysis or visualization.

The SQLite database (Supplementary Files) is finally queried to get the generated peptide sequences. Identical peptide sequences are aggregated, and the accession identifiers of all source sequences are concatenated into a semicolon-delimited annotation field. This result and summary statistics (e.g. length of the peptide sequence, number of genes) are reported in the final table and delivered to the user as a comma separated values (CSV) table (Table 1). In addition to the table, PEPTiGEN outputs FASTA files with all the sequences described above and the multiple sequence alignments. The interface to the SQLite database is implemented in Python v3.8.20 using SQLAlchemy v1.3.13 (see Fig. 1).

**Table 1:** Part of PEPTiGEN output of generated peptides using the blaCTX-M-15 reference sequence as input.

Predicted peptide sequence^a^	Length^b^	GenBank accessions analysed (n)^c^	Present in GenBank accessions (n)^d^	GenBank accessions IDs^e^
RLGVALINTADNSQILYR	18	4998	4950	AP023226, AP023225, …
RFAMCSTSK	9	4998	4944	AP023226, AP023225, …
KALGDSQR	8	4998	4941	AP023226, AP023225, …
RAMAQTLR	8	4998	4939	AP023226, AP023225, …
RQLGDETFR	9	4998	4936	AP023226, AP023225, …
KKSDLVNYNPIAEK	14	4998	4932	AP023226, AP023225, …
RTEPTLNTAIPGDPR	15	4998	4897	AP023226, AP023225, …
RAPLILVTYFTQPQPK	16	4998	4840	AP023226, AP023225, …
KVMAAAAVLKK*	11	4998	3693	AP023226, AP023225, …
KVMAAAAVLK*	10	4998	3693	AP023226, AP023225, …
KVMAVAAVLKK*	11	4998	1255	CP055257, CP055248, …
KVMAVAAVLK*	10	4998	1255	CP055257, CP055248, …
KAIPPVQR	8	4998	22	CP036188, CP034137, …

Values show output of PEPTiGEN using the preset BLAST thresholds of 80% for coverage and 95% for identity, thereby selecting all closely related *bla*_CTX-M-15_ variants part of the *bla*_CTX-M-1_ group.

a Though not part of tryptic peptides *in vitro*, C-terminal Arginine (R) and Lysine (K) residues of the previous peptide are included in the sequence.

b Number of amino acid moieties in peptide sequence as predicted by PEPTiGEN.

c Total number of GenBank accessions analysed.

d Number of GenBank accessions in which the peptide was detected.

e GenBank accession ID’s in which the peptide was identified.

* All possible tryptic peptides are generated to account for variations in both amino acids and ragged ends.

### 2.3 Portability and software management

PEPTiGEN is implemented as a Snakemake workflow. As such, it supports execution on a range of different infrastructures, enabling parallel and multi-threaded job execution where possible. Users are required to configure execution settings depending on their computing platform. PEPTiGEN includes example configuration files for both single-server and distributed computing infrastructures.

PEPTiGEN leverages Snakemake’s integration with the Conda package manager. Each required tool is installed in a dedicated Conda environment, and PEPTiGEN is shipped together with environment definition files to facilitate installation and software management. In addition, all Conda environments are encapsulated in a Docker container, which can be automatically downloaded and managed via the Snakemake command-line interface simplifying software environment management.

### 2.4 Validation of PEPTiGEN peptide output

The peptide output of PEPTiGEN was first validated *in silico* by comparing predicted peptides for 13 different AMR genes with manually curated peptides, generated using a previously described method (Foudraine *et al.* 2019). Briefly, a BLASTn search was performed to include all available full-length or near full-length variants of each gene. BLAST thresholds were selected per gene by examining the coverage and identity of the hits: variants above a natural gap in these metrics were included, while more distant homologs below the gap were excluded, typically resulting in thresholds around 90% coverage and 95% identity. DNA sequences were translated to proteins, aligned using ClustalX (Larkin *et al.* 2007) and digested *in silico* with trypsin. For each peptide in a range of 6–20 amino acid moieties with possible multiple variants, the variant present in most GenBank accessions was selected.

PEPTiGEN output was further validated using peptides previously detected in vitro. Peptide sequences were retrieved from publications by Charretier *et al.* (2015a), Foudraine *et al.* (2019), Wang *et al.* (2019a, 2019b), Lu *et al.* (2022) and Deforet *et al.* (2024). These studies provided in vitro evidence for AMR-peptides. We verified the presence of these published AMR peptides in the PEPTiGEN output using the corresponding reference sequences from the Comprehensive Antimicrobial Resistance Database (CARD) (Alcock *et al.* 2023) as input.

### 2.5 In silico creation of AMR peptide database

For in-depth testing of PEPTiGEN, we analysed all AMR genes from the externally curated CARD database, thereby running PEPTiGEN 4839 times individually. As the CARD database curates AMR genes as individual genes (e.g. blaSHV-1 and blaSHV-2), PEPTiGEN was run using a BLAST coverage threshold of 80% and a BLAST identity threshold of 95%. These parameters provide a reproducible balance between specificity and peptide recovery. For the construction of the database, a specific version of PEPTiGEN was developed that omits the Clustal Omega analysis to reduce the computational time of the analysis. For the analysis of all CARD genes with PEPTiGEN, the nt_prok database was downloaded in September 2024.

After running PEPTiGEN 4839 times individually, all results were used to construct the AMR peptide database, which was stored as a SQL database (Supplementary Files). The database was created by merging all results from PEPTiGEN using the CARD Antibiotic Resistance ontology (ARO) index, enabling queries by peptide sequence as well as by occurrences. Further downstream analysis of the peptides of this database was performed using RStudio version 4.3.2.

## 3 Results

PEPTiGEN is a computational workflow compatible with any Linux-based computer [local PCs, servers, virtual machines (VM)]. The workflow utilizes widely adopted tools, including BLAST (Altschul *et al.* 1990). The input is a nucleotide sequence of a prokaryotic resistance gene, which PEPTiGEN then uses as a query against the NCBI GenBank nt_prok database (Benson *et al.* 2013). This process retrieves all sequence variants from the BLAST database. Subsequently, sequences are translated, and tryptic peptides representing all variants of the AMR gene are generated (Fig. 1). To optimize detection potential by LC-MS/MS, the resulting tryptic peptides are filtered to include only those sequences that are 6–20 amino acids in length and contain no missed cleavages. To retain relevant information of ragged C- and N-termini, the C-terminal Arginine (R) and Lysine (K) residues of the preceding peptide are included in the peptide sequence within the database, even though they are not part of tryptic peptides in vitro. For example, Table 1 shows part of the actual CSV output of all generated tryptic peptides for the reference sequence of the AMR-gene *bla*_CTX-M-15_. Since PEPTiGEN generates all possible tryptic outputs, both K.VMAAAAVLK and K.VMAAAAVLKK are generated to account for variations in ragged ends.

PEPTiGEN produces multiple outputs including all unique protein variants, all generated tryptic peptides, and BLAST query details, which are stored in an accessible, interoperable, and user-friendly SQLite database. This database is enriched by metadata and statistical data, such as the GenBank accession IDs of all sequences that met the BLAST thresholds, the primary sequence position of each peptide generated from a protein, and the GenBank accession IDs of the sequences in which each peptide is detected. Depending on the version of the tool used, PEPTiGEN also outputs the alignments of all nucleotide and protein sequences. On average, running a single complete analysis from sequence input to peptide output takes 14 minutes, with a broad range spanning from 1 to 40 minutes (Fig. S1). The run time depends on factors such as the identity threshold of the BLAST analysis, the maximum number of genes retrieved, and the number of cores available on the virtual machines, local PC or server.

### 3.1 Validation of PEPTiGEN peptide output

To validate the peptides generated by PEPTiGEN, we compared its output with both *in silico* generated AMR peptides and published *in vitro* AMR peptides. First, we compared the PEPTiGEN output for 13 AMR genes with manually curated peptides (Table 2(A)). These were generated using a BLASTn search, selecting full-length and near-full-length sequences, translating them, and simulating tryptic digestion, following the method by Foudraine *et al.* (2019). The manual procedure generated 226 peptide sequences, of which PEPTiGEN generated 99% (224 out of 226, Table 2(A)). The two missing peptides were derived from the aminoglycoside-modifying enzyme aad(6). Further analysis revealed that the reference sequence in CARD used as input lacked 26 residues at the N-terminus, explaining the discrepancy. When a complete aad(6) sequence from the NCBI Reference Gene Catalog (Goldfarb *et al.* 2025) (NG_047393.1) was provided, PEPTiGEN generated all predicted peptides.

**Table 2:** *In silico* (A) and *in vitro* (B) validation of PEPTiGEN peptide output.

A		B		
AMR Gene	PEPTiGEN generated peptides/manually curated peptides (%)	AMR Gene	PEPTiGEN generated peptides/literature affirmed peptides (%)	Reference
aac(6′)-Ie-aph(2″)-Ia	26/26 (100%)	aac(6′)-Ie-aph(2″)-Ia	3/3 (100%)	Deforet *et al.* (2024)
aad6^a^	13/15 (87%)	ant(4′)-I^b^	2/3 (67.7%)	Deforet *et al.* (2024)
ant(6′)-Ia	16/16 (100%)	aph(3′)-III	3/3 (100%)	Deforet *et al.* (2024)
ant(4′)-Ia	4/4 (100%)	CMY-2	2/2 (100%)	Lu *et al.* (2022)
aph(3′)-IIIa	12/12 (100%)	CTX-M	5/5 (100%)	Lu *et al.* (2022)
blaZ	14/14 (100%)	IMP	4/4 (100%)	Lu *et al.* (2022)
ermA	15/15 (100%)	KPC	5/5 (100%)	Foudraine *et al.* (2019), Lu *et al.* (2022)
ermC	10/10 (100%)	MCR-1	3/3 (100%)	Wang *et al.* (2019a)
mecA	33/33 (100%)	NDM	6/6 (100%)	Foudraine *et al.* (2019), Wang *et al.* (2019b), Lu *et al.* (2022)
mecC	39/39 (100%)	OXA-1	6/6 (100%)	Lu *et al.* (2022)
vanA	18/18 (100%)	OXA-48	2/2 (100%)	Foudraine *et al.* (2019)
vanB	15/15 (100%)	mecA	8/8 (100%)	Charretier *et al.* (2015a), Deforet *et al.* (2024)
vanC	9/9 (100%)	mecC	4/4 (100%)	Charretier *et al.* (2015a), Deforet *et al.* (2024)
		TEM	1/1 (100%)	Lu *et al.* (2022)
		VIM	4/4 (100%)	Foudraine *et al.* (2019), Lu *et al.* (2022)

Values represent the proportion of (A) all manually generated peptides of the protein based on genome sequence, and (B) experimentally detected peptides published in literature, that are generated by PEPTiGEN using the CARD reference sequence of the specified AMR gene as input.

a Incomplete CARD reference sequence.

b GTG start codon.

Next, we compared PEPTiGEN’s output with *in vitro* LC-MS/MS results from six publications about experimental validations of targeted LC-MS/MS assays for the detection of AMR proteins. These included one of our previous studies (Foudraine *et al.* 2019) and five studies from other research groups (Charretier *et al.* 2015a, Wang *et al.* 2019a, 2019b, Lu *et al.* 2022, Deforet *et al.* 2024). Together, the six publications reported 59 AMR peptides. PEPTiGEN correctly generated 58 of these 59 peptides (98.3%, Table 2(B)). The only exception was the N-terminal peptide MNGPIIMTR from ant(4′)-Ia detected by Deforet *et al.* (2024), which was not generated due to the use of an alternative start codon (GTG). As PEPTiGEN only accounts for ATG start codons, this class of peptides is missed.

Finally, we assessed PEPTiGEN’s ability to detect other bacterial peptides, specifically virulence-associated peptides. Charretier *et al.* (2015a) identified peptides of the virulence genes toxic shock syndrome toxin 1 (TSST-1) and the Panton-Valentine Leukocidin (PVL) subunits LukF and LukS using LC-MS/MS. PEPTiGEN generated identical TSST-1, LukF, and LukS peptides when using their respective gene sequences as input. This supports the extensibility of the tool and its use beyond the generation of AMR peptides of bacterial origin.

### 3.2 Identification of conserved peptides for AMR genes using PEPTiGEN’s peptide output

PEPTiGEN’s peptide output enables identification of conserved peptides across AMR gene variants under the predefined BLAST thresholds. By using metadata on the number of GenBank accessions analysed per AMR gene and peptide occurrence across these accessions, peptide conservation can be determined. For example, in Table 1, the peptide RLGVALINTADNSQILY is present in 4950 of the 4998 analysed *bla*_CTX-M-15-like_ GenBank accessions, whereas peptide KVMAVAAVLK is present in only 1255. With BLAST thresholds of 80% for coverage and 95% for identity and *bla*_CTX-M-15_ as input, PEPTiGEN selects all closely related *bla*_CTX-M_ variants belonging to the *bla*_CTX-M-1_ group. Thus, by using only *bla*_CTX-M-15_ as input, PEPTiGEN generates information on conserved peptides across the entire CTX-M-1-like group (Table 1). Conserved peptides in other CTX-M-groups, such as CTX-M-2-like or CTX-M-9-like, can be generated using their respective reference sequences, while lowering the identity threshold enables recovery of peptides conserved across the entire CTX-M-family.

### 3.3 AMR gene peptide database for direct MS application

We demonstrated that PEPTiGEN detects experimentally resolved AMR peptides from bacteria, as well as peptides from virulence-associated genes, and provides insights into conserved peptides. While conserved peptides are essential for developing targeted LC-MS/MS assays, additional information on peptide specificity for individual AMR genes or gene families is required. To address this need, we applied PEPTiGEN to all known AMR-associated gene sequences to build the first AMR peptide database, enabling simultaneous assessment of peptide conservation and gene-family specificity to support targeted design. We ran PEPTiGEN automatically on 4839 gene sequences from the CARD database (downloaded September 2024) and annotated the output using the CARD ontology.

Overall, PEPTiGEN generated results for 4606 CARD reference sequences (95.2%) processing 86 198 different GenBank accessions. The complete AMR peptide database contains a total of 667 861 peptides, representing 73 791 different peptide sequences after removal of duplicates (Table S1).

**Figure 2:**
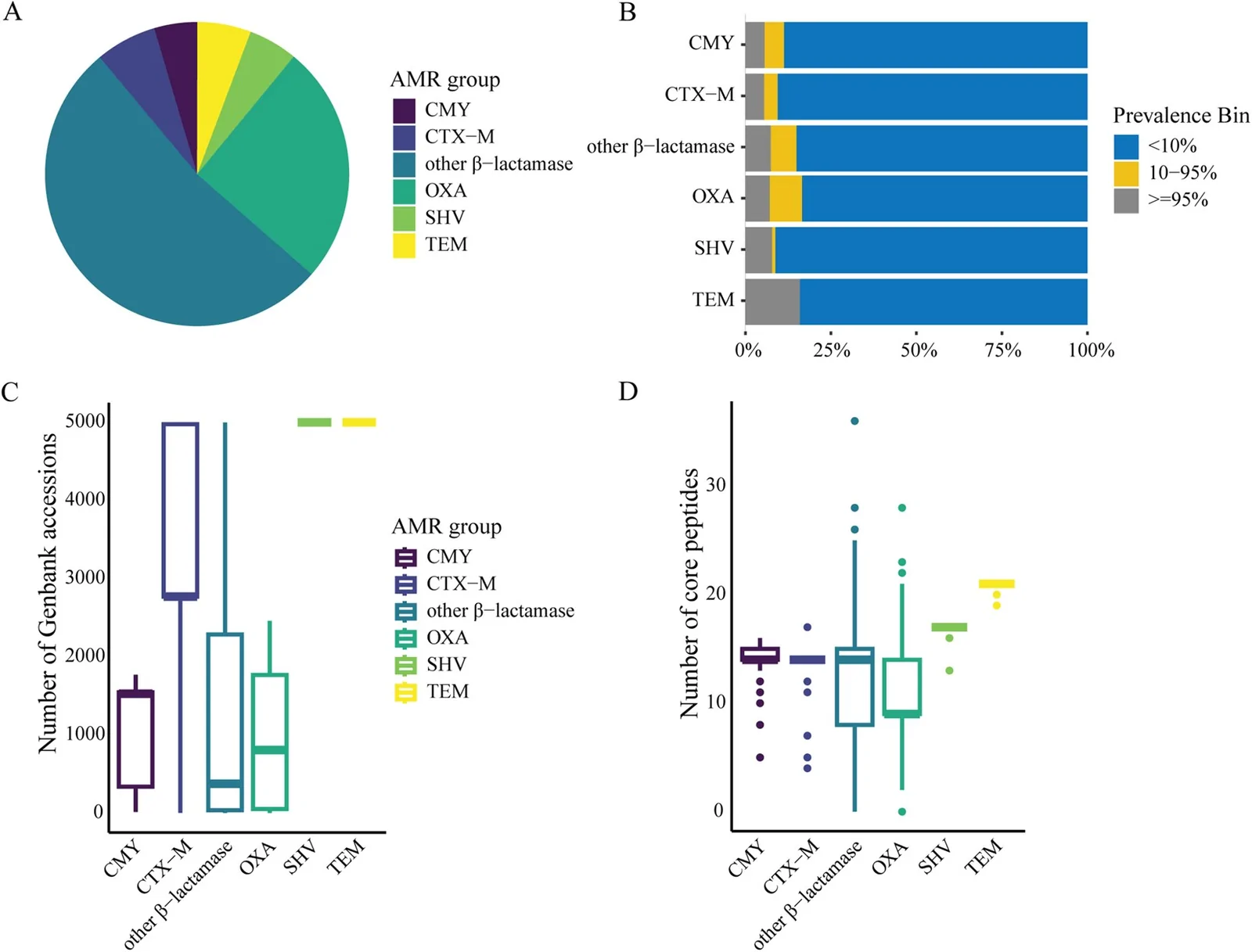
Characteristics of peptides in AMR peptide database across β-lactamase groups. (A) Distribution of the number of AMR genes across the six β-lactamase groups. (B) Percentage of peptides present in <10%, 10–95%, and ≥95% (core peptides) in each β-lactamase group. (C) Boxplots representing the number of analysed GenBank accessions per AMR gene and (D) the number of core peptides per AMR gene, categorized by β-lactamase group.

To demonstrate the utility of the AMR peptide database in selecting peptides unique for AMR-genes or AMR-gene families, we focused on peptides derived from β-lactamase genes. All CARD β-lactamase gene families and corresponding CARD reference sequences were arbitrarily categorized into 6 AMR groups; CMY β-lactamases, CTX-M β-lactamases, OXA β-lactamases, SHV β-lactamases, TEM β-lactamases and remaining other β-lactamases (Fig. 2A, Table S2). Each β-lactamase gene was aligned to a median number of 849 GenBank entries (IQR: 82–2285) and generated a median of 128 peptides (IQR: 49–272; Table S3). In result, the database contains 582 332 peptides of 3501 β-lactamase genes, of which 15 942 different peptide sequences (Table S1).

Most peptides (502 600 of 582 332; 86%) occur in <10% of the analysed GenBank accessions for each gene, highlighting their non-conserved nature (Fig. 2B). In contrast, only 43 533 peptides (7.5%) are detected in >95% of the analysed GenBank accessions for each gene. This highly conserved subset, termed “core peptides,” represents promising candidates for targeted LC-MS/MS assays. The median number of core peptides per β-lactamase gene was 14 (IQR: 9–15; Table S3), with at least one core peptide identified for 98.8% of β-lactamase genes (3458 of 3501).

**Figure 3:**
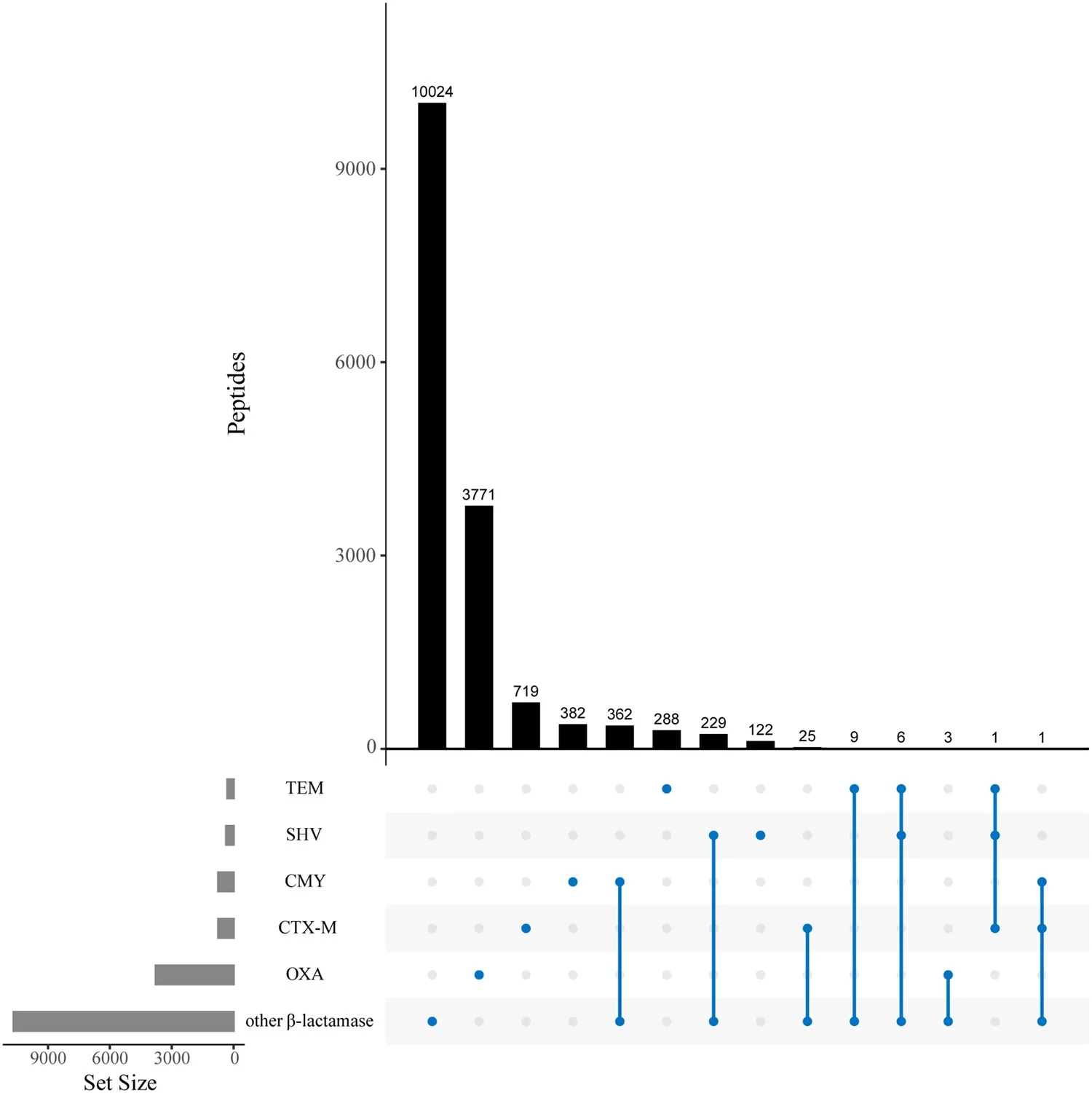
Upset plot of all β-lactamase peptides of the AMR peptide database with their level of specificity for the different β-lactamase groups. The vertical bar plots (black), plotted in descending order, represent the total number of peptides generated for a single or multiple β-lactamase groups, as represented in the bottom by dots (blue). The horizontal bar plots (grey) show the total number of peptides generated for each β-lactamase group.

**Figure 4:**
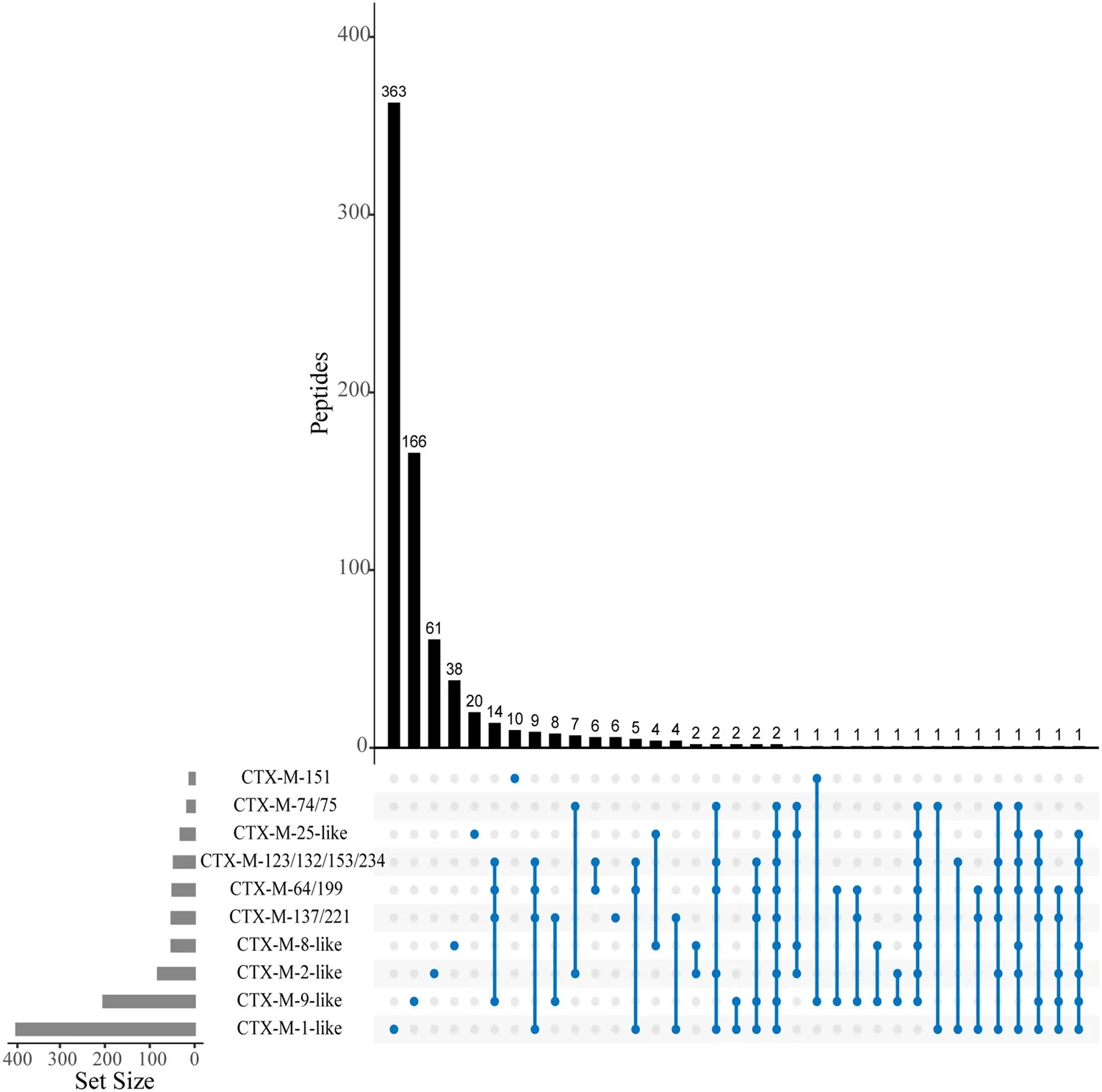
Upset plot of all CTX-M peptides of the AMR peptide database with their level of specificity for the different CTX-M subgroups. The vertical bar plots (black), plotted in descending order, represent the total number of peptides generated for a single or multiple CTX-M subgroups, as represented in the bottom by dots (blue). The horizontal bar plots (grey) show the total number of peptides generated for each CTX-M subgroup. The CTX-M subgroups are classified based on 95% identity on genetic level.

The AMR peptide database enables rapid analysis of peptide overlap across AMR gene groups. The upset plot in Fig. 3 shows that most β-lactamase peptides are group-specific, while also highlighting the peptides shared between groups. For example, 719 peptides are unique to the CTX-M family, whereas only 25 are shared with the “other β-lactamase” group, suggesting high group-level specificity relevant for LC-MS/MS assay design. Of these 25 shared CTX-M peptides, most overlap with OXY, RAHN, and FONA-β-lactamases. The upset plot in Fig. 4 shows more extensive peptide sharing between CTX-M subgroups, defined by 95% genetic identity percentage and literature-based grouping (D’Andrea *et al.* 2013), reflecting the higher sequence similarity between these subgroups. Nevertheless, subgroup-specific peptides were identified, indicating that peptide-level discrimination between CTX-M subgroups is possible. In essence, the AMR peptide database enables the identification of peptides unique to specific AMR-genes or gene families, supporting the design of targeted LC-MS/MS assays and diagnostic applications.

### 3.4 Enabling in-depth analysis of AMR gene families

The AMR peptide database also enables more in-depth analysis of specific AMR groups; for instance, for all AMR genes of both the SHV β-lactamase group and the TEM β-lactamase group, genes from 4995 to 5000 different GenBank accessions were analysed, respectively. However, 99% (182 of 183) of the SHV β-lactamase genes and 100% (199 of 199) of the TEM β-lactamase genes have > 15 core peptides. This suggests the presence of large, highly conserved protein regions, despite wide distribution of the genes (Fig. 2C and D).

## 4 Discussion

Accurate and rapid diagnostic methods for antimicrobial resistance testing are crucial in facilitating early adequate therapy, especially as the global rise in AMR threatens the efficacy of antibiotics (Bassetti *et al.* 2022, GBD Antimicrobial Resistance Collaborators 2024). One recently introduced and validated method for rapid AMR detection is LC-MS/MS (Aldea *et al.* 2025), which may decrease time-to-result with 18–48 hours. Successful implementation of targeted mass spectrometry approaches requires comprehensive peptide-level information linked to AMR mechanisms, as over 4500 AMR mechanisms have been documented to date (Alcock *et al.* 2023). We present an open-source tool that automatically generates up-to-date tryptic peptides for any AMR gene, reducing the need for manual processing of individual resistance mechanisms for LC-MS/MS assay design. The tool was validated by comparing its output to both manual processed AMR peptides and published AMR peptides from validated LC-MS/MS assays for AMR detection. Subsequently, we used PEPTiGEN to accomplish the construction of the first AMR peptide database.

The key features of PEPTiGEN are the ability to generate AMR peptides, validated both *in silico* and *in vitro*, and to produce multiple output formats, including peptide occurrence metadata from public repositories. This enables rapid differentiation between conserved and variable peptides, which is particularly useful for AMR genes with limited official subvariant annotation such as *mecA* and *mecC*. Outputs can be optimized to different scenarios by adjusting BLAST thresholds per run. In addition, the aligned nucleotide output may assist in selecting conserved regions for PCR assay design. The short computational time of minutes per AMR gene and containerized design facilitate rapid updating after new GenBank or CARD releases. With further validation, PEPTiGEN may also be applied to any other prokaryotic mechanism of interest, as shown for the bacterial virulence genes *PVL* and *TSST-1*.

The AMR peptide database opens new opportunities for clinical applications and research by cataloguing all AMR peptide variants and providing information on both peptide specificity and variability. Peptides unique to an AMR gene facilitate targeted detection, while variability highlights conserved and divergent regions, relevant for assay design and evolutionary analyses. With this information, the database facilitates LC-MS/MS method development and provides a foundation for further studies on AMR genes and mechanisms. For example, our analysis suggested large conserved protein regions of TEM and SHV β-lactamase genes, consistent with previous reports (Liakopoulos *et al.* 2016, Castanheira *et al.* 2021). The database’s metadata can also be used to link peptide sequences to public records and support downstream computational, phylogenetic, or functional analyses. With additional information on bacterial species and country of sequencing from NCBI, this may further enable the study of distribution and evolution of AMR genes across species and geographic regions. The AMR peptide database is available as an SQL-database and is additionally provided as a CSV file containing all GenBank accession IDs and generated peptides (see Data availability).

While PEPTiGEN and the AMR peptide database are a great improvement for implementing LC-MS/MS in AMR diagnostics and research, several limitations should be considered. First, PEPTiGEN generates all theoretical tryptic peptides, including ragged termini. This lead to overrepresentation of such “ragged” peptides in both the output and the database compared to experimental observations. Although all theoretical peptides of MS-compatible length are generated, not all peptides are necessarily suitable for LC–MS/MS analysis due to physicochemical properties. Additional filtering using complementary tools to predict peptide properties may be required to prioritize candidates for targeted MS workflows. In addition, PEPTiGEN currently performs best for horizontally acquired AMR genes, where resistance is determined by protein presence or absence, and for chromosomal genes that confer resistance through upregulated or downregulated protein expression. In contrast, it is less directly suited for resistance driven by specific mutations or loss-of-function frameshifts. Known mutations should first be mapped back to generated peptides, while frameshift-related resistance requires an additional preprocessing step to separate frameshifted sequences from canonical sequences prior to peptide extraction.

For the AMR peptide database, some additional limitations should be considered. First, the database was generated using BLAST thresholds of 80% coverage and 95% identity, balancing specificity and peptide recovery. However, optimal thresholds are likely to vary across AMR gene families, and less stringent or group-specific settings may improve peptide recovery as enabled by PEPTiGEN. Second, the AMR peptide database was built using the CARD ontology, a well-curated, peer-reviewed resource; however, an alternative ontology may be applied for specific research questions.

Finally, both PEPTiGEN and the AMR peptide database are computational tools to support targeted LC-MS/MS assay design. Experimental performance depends on MS acquisition mode, peptide properties, and biological variation in AMR expression, which may be influenced by conditions such as antibiotic exposure. These aspect should be considered in study-specific experimental design.

In conclusion, we have developed an open source and user-friendly tool, which quickly generates accurate peptides for any AMR gene. The use of PEPTiGEN omits the laborious and time-consuming task of manually generating peptide information. Subsequently, we have created the very first AMR peptide database. The combination of both PEPTiGEN and the AMR peptide database sets a solid foundation for pioneering applications of LC-MS/MS in research and clinical diagnostics.

## Supplementary Material

btag604_Supplementary_Data

## Data Availability

The PEPTiGEN code and AMR peptide database are publicly available at github (https://github.com/ftabaro/inspection) and Zenodo (https://doi.org/10.5281/zenodo.21196702).
