# Supplementary material for: PEPTiGEN: a tool for mining antimicrobial resistance PEPTides using GENe data of public available repositories
Source: Bioinformatics. 2026 Aug 18;42(8):btag604. doi: 10.1093/bioinformatics/btag604 (PMC13505617; doi:10.1093/bioinformatics/btag604)
Supplement: Supplementary file 1 [file btag604_supplementary_data.zip › Supplementary Files.pdf]

Supplementary Files

SQL database schema AMR Peptide database

Tables

Peptides

One row per unique (CARD gene, peptide sequence) combination, with prevalence already computed.

Column	Type	Description
**`id`**	`INTEGER, PK`	Unique peptide-record identifier
**`ARO_Accession`**	`TEXT`	CARD ARO accession (ARO:NNNNNNN), links to AroIndex
**`sequence`**	`TEXT`	Peptide amino acid sequence
**`length`**	`INTEGER`	Peptide length (residues)
**`ARO_Name`**	`TEXT`	CARD gene/ontology name
**`AMR_Gene_Family`**	`TEXT`	AMR gene family (e.g. "CTX-M beta-lactamase")
**`AMR_Group`**	`TEXT`	Higher-level AMR group used for grouping figures
**`n_Genbank`**	`INTEGER`	Number of distinct GenBank accessions this peptide was found in, for this gene
**`Total_Genbank_per_ARO`**	`INTEGER`	Total distinct GenBank accessions analyzed for this gene
**`prevalence`**	`REAL`	100 * n_Genbank / Total_Genbank_per_ARO — the % of this gene's variants that contain this peptide

Genes

Distinct GenBank accessions (BLAST hit subject accessions) across the whole database.

Column	Type	Description
**`id`**	`INTEGER, PK`	
**`gene_name`**	`TEXT`	GenBank accession

PeptideGenes

Many-to-many join table: every individual GenBank accession each peptide record was found in (119,353,976 rows — this is the full accession-level detail; `Peptides.n_Genbank` is just its count).

Column	Type	Description
**`peptide_id`**	`INTEGER, FK`	→ Peptides.id
**`gene_id`**	`INTEGER, FK`	→ Genes.id

AroIndex

One row per CARD gene analyzed, with its AMR annotation.

Column	Type	Description
**`ARO_Accession`**	TEXT	CARD ARO accession (joins to Peptides.ARO_Accession)
**`ARO_Name`**	TEXT	Gene/ontology name
**`AMR_Gene_Family`**	TEXT	AMR gene family
**`AMR_Group`**	TEXT	Higher-level AMR group

Relationships

```
AroIndex (1) —< (many) Peptides
Peptides (1) —< (many) PeptideGenes >— (1) Genes
```

Typical queries

Every GenBank accession a given peptide occurs in, with CARD annotation:

```
SELECT p.ARO_Name, p.sequence, g.gene_name, p.prevalence
FROM Peptides p
JOIN PeptideGenes pg ON pg.peptide_id = p.id
JOIN Genes g ON g.id = pg.gene_id
WHERE p.sequence = 'RLGVALINTADNSQILYR'
```

Core (highly conserved, ≥95% prevalence) peptides for a gene family:

```
SELECT sequence, ARO_Name, n_Genbank, Total_Genbank_per_ARO, prevalence
FROM Peptides
WHERE AMR_Gene_Family = 'CTX-M beta-lactamase' AND prevalence >= 95
ORDER BY prevalence DESC
```

SQL database schema PEPTiGEN

Tables

genbank_sequences

Metadata for GenBank nucleotide records, including accession number, title, organism, and sequence length.

Column	Type	Description
**id**	INTEGER, PK	
**genbank_id**	STRING	GenBank accession ID
**accession**	STRING	BLAST hit accession
**sequence_length**	INTEGER	Sequence length
**title**	STRING	BLAST hit title
**organism**	STRING	BLAST hit organism

blast_hits

BLAST alignment results associated with GenBank records, including alignment statistics, coordinates, reading frame, and translated protein sequence when available.

Column	Type	Description
**id**	INTEGER, PK	
**qacc**	STRING	Query accession
**sacc**	STRING	Subject accession
**nucl_seq**	STRING	Nucleotide sequence
**prot_seq**	STRING	Protein sequence
**coverage**	FLOAT	Query coverage
**percent_identity**	FLOAT	Query percentage identity
**qstart**	INTEGER	First aligned nucleotide from query
**qend**	INTEGER	Last aligned nucleotide from query
**sstart**	INTEGER	First aligned nucleotide from subject
**send**	INTEGER	Last aligned nucleotide from subject
**sframe**	INTEGER	Subject frame
**bitscore**	FLOAT	bit-score
**evalue**	FLOAT	e-value
**translated**	BOOLEAN	
**genbank_id**	INTEGER, FK	→ genbank_sequences.id

peptides_instances

Occurrences of peptides within BLAST hits, recording the peptide identity and its start/end coordinates relative to the hit sequence.

Column	Type	Description
**id**	INTEGER, PK	
**relative_start**	INTEGER	Start in aligned sequence
**relative_end**	INTEGER	End in aligned sequence
**hit_id**	INTEGER, FK	→ blast_hits.id
**peptide_id**	INTEGER, FK	→ peptides.id

peptides

Unique peptide sequences identified in the analysis.

Column	Type	Description
**id**	INTEGER, PK	
**sequence**	STRING	Peptide amino acid sequence

Relationships

```
genbank_sequences (1) ──< (many) blast_hits
blast_hits (1) ──< (many) peptides_instances >── (1) peptides
```

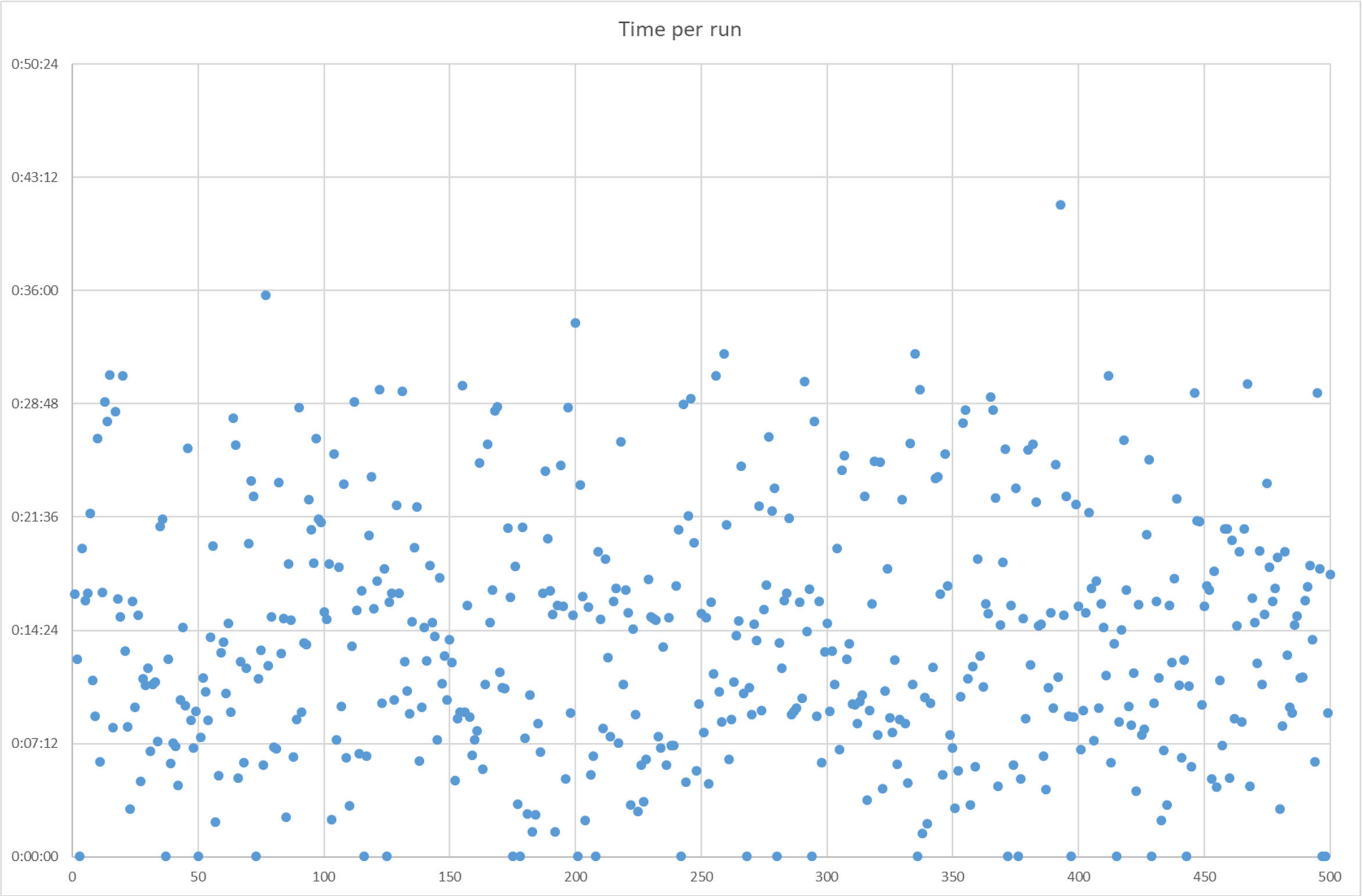

Supplementary Figure 1. Run time of PEPTiGEN for a randomly selected subset of 500 CARD reference sequences. The x-axis represents the different CARD reference sequences, while the y-axis indicates run time in minutes.

Supplementary Table 1. Analysis Overview of the CARD-database using PEPTiGEN.

	All AMR genes	β-lactamase genes
**AMR genes**	4,606	3,501
**GenBank IDs analyzed**	86,198	52,504
**AMR gene families**	311	219
**Peptides**	667,861	582,332
**Distinct peptide sequences~**	73,791	15,942
**Unique peptides***	58,118	5,072

~ the total number of peptides in the non-redundant version of the database; * the number of peptides derived exclusively from a single AMR gene

Supplementary Table 3. Characteristics of peptides present in AMR peptide database

Statistic	All #	TEM	SHV	OXA	CMY	CTX-M	other β-lactamase
**AMR genes; n (%)***	4,606	199 (4.3%)	183 (4.0%)	892 (19.4%)	161 (3.5%)	228 (5.0%)	1,838 (40.0%)
**Peptides; n (%)**	667,861	26,163 (3.9%)	39,638 (5.9%)	129,063 (19.3%)	39,960 (6.0%)	54,219 (8.1%)	296,289 (44.4%)
**GenBank accessions analyzed; n**	86,198	7,658	5,575	11,983	2,057	8,333	35,343
**AMR gene families; n**	311	1	1	2	1	1	213
**Peptides generated per AMR gene**	103 (34 – 254)	137 (107 – 152)	216 (202 – 226)	111 (52 – 263)	315 (124 – 334)	201 (201 – 349)	85 (34 – 161)
**Core peptides per AMR gene****	13 (9 – 15)	21 (21 – 21)	17 (17 – 17)	9 (9 – 14)	14 (14 – 15)	14 (14 – 14)	14 (8 – 15)
**GenBank accessions analyzed per AMR gene**	517 (40 – 2285)	5000 (5000 – 5000)	4999 (4999 – 4999)	807 (53 – 1768)	1527 (337 – 1545)	2770 (2770 – 4976)	376 (36 – 2285)

Generated peptides for all genes in CARD database, including β-lactamase genes, AME genes, and other AMR-related genes ; * number of AMR genes that generated results; ** core peptide = peptide present in >=95% of all GenBank accessions analyzed for the AMR gene.
Values represent median (interquartile range ((IQR)), unless otherwise stated.
